# Effect of the Covid 19 pandemic on depression and mother-infant bonding in uninfected postpartum women in a rural region

**DOI:** 10.1186/s12884-022-04580-8

**Published:** 2022-03-19

**Authors:** Özlem Erten, İsmail Biyik, Cenk Soysal, Onur Ince, Nadi Keskin, Yasemin Tascı

**Affiliations:** 1 Department of Gynecology and Obstetrics, Faculty of Medicine, Kutahya Health Sciences University, Kutahya, Turkey; 2 Department of Statistics, Faculty of Arts and Science, Middle East Technical University, Ankara, Turkey

**Keywords:** Postpartum depression, Maternal-infant bonding, COVID 19 pandemic, Visitor restriction

## Abstract

**Background:**

Postpartum depression and maternal-infant attachment scores were examined in uninfected women during the COVID 19 pandemic in Kutahya, a rural province in Turkey's North Aegean region.

**Methods:**

This cohort study was conducted in the Kutahya Health Sciences University Hospital obstetrics unit between April 2021 and August 2021. 178 low-risk term pregnant women who gave birth were given the surveys Edinburgh Postpartum Depression Scale and Mother-to-Infant Bonding Scale (MIBQ) 6 weeks after birth. The Edinburgh Postpartum Depression Scale was used to determine postpartum depression and the Mother-to-Infant Bonding Scale was used to determine maternal attachment.

**Results:**

In this study, the postpartum depression rate was calculated as 17.4%. When depressed and non-depressed patients were compared, education level, maternal age, BMI, MIBQ score, history of previous pregnancies, route of delivery, previous operation history, economic status, employment status and pregnancy follow-up information were found to be similar (p > 0.05). The ratings on the Mother-to-Infant Bonding Scale were found to be similar in depressed and non-depressed patients (p > 0.05). The odds of maternal depression for patients who received guests at home was 3.068 (95%CI [1.149–8.191]) times the odds of patients who did not receive guests at home.

**Conclusions:**

Although a relationship has been found between accepting guests in the postpartum period and postpartum depression, it is necessary to investigate in further studies whether there is a causal relationship.

## Introduction

Giving birth is a very special experience for mothers. Almost all postpartum women welcome birth with joy. However, some puerperant women exhibit postpartum depressive symptoms [1]. Although several criteria exist for postpartum depression (PPD), unipolar depression that arises within 12 months of birth is widely considered as postpartum depression [2]. The prevalence of postpartum depression has been reported as 9% [3]. The prevalence of postpartum depression varies depending on the society in which the study is conducted, the sociological structure of the society, income level and time since giving birth [4, 5].

Risk factors for postpartum depression are women’s age, high life stress, lack of social support, current or past abuse, prenatal depression, marital or partner dissatisfaction, and some clinical delivery difficulties [2].

Maternal attachment is defined as a mother's emotional state that displays a mother's thoughts and cognitions toward her baby and herself as a parent [6, 7]. Attachment behaviour emerges during pregnancy or immediately after birth and continues throughout the infant's first months [6, 8]. Postpartum depression may negatively affect maternal-infant attachment [9, 10]. Negative maternal-infant bonding effects caused by depression may result in newborn malnutrition and poor health outcomes [10–12].

After the coronavirus pandemic started, the first case in Turkey was reported on March 11, 2020, and the first death was reported on March 17, 2020. On March 23, 2020, it was announced that the COVID 19 pandemic had spread to the whole country of Turkey (https://covid19.saglik.gov.tr). COVID-19 infection is associated with increased maternal and fetal risks [13]. During the COVID 19 pandemic period, postpartum depression rates increased and mother attachment scores fell among pregnant women in the early postpartum period, according to a preliminary study conducted in Turkey [14]. Similarly, during the COVID 19 pandemic, maternal nervous and depression symptoms increased, and mother to -infant bonding was reduced, according to studies conducted in various societies [15–17]. However, there are also studies reporting that although the severity of postpartum depression increased during the COVID 19 pandemic, maternal-infant attachment was not affected [18–20]. In Australian studies, although puerperal women experienced stress due to the COVID 19 pandemic, they reported that the pandemic period, being alone, and restrictions, had positive aspects such as mothers spending more time with their babies, more rest, more time for breastfeeding and more time for attachment to their baby [19, 20].

In this study, depression and maternal-infant attachment scores were explored among uninfected women during the COVID 19 pandemic in Kutahya, a rural province in Turkey's Aegean area.

## Materials and methods

### Study design and participants

This cohort study was conducted in the Kutahya Health Sciences University Hospital obstetrics and gynecology out-patient unit between April 2021 and August 2021. Ethical approval was obtained from the Ethics Committee of Kutahya Health Sciences University (Research number: 2021/07–09), Kutahya, Turkey. Informed consent was obtained from all women included in the study. In our hospital, visitor restriction and prohibition policies were applied for delivery and postpartum units during the pandemic. Those who delivered vaginally were discharged after 24 h, while those who delivered by cesarean section were discharged 48 h later. They were advised to limit visitors and consider celebrating the birth of the baby using phone or online tools.

One hundred seventy-eight low-risk term pregnant women who gave birth were given the surveys Edinburgh Postpartum Depression Scale and MIBQ scale 6 weeks after birth. Those with high-risk pregnancies such as multiple pregnancies, preterm birth, fetal growth retardation, fetal anomaly, preeclampsia, stillbirth, autoimmune disease, chronic disease with vascular involvement, neurological or psychiatric disease, history of previous postpartum depression and those who did not understand Turkish were not included in the study. All the patients were contacted by phone. Phone calls were made by OE, an obstetrician and gynecologist. Postpartum women were informed about the study. Their consent was taken. Then, the questionnaire questions and demographic information were filled. Demographic data, birth information, Edinburgh Postpartum Depression Scale, and Mother-to-Infant Bonding Scale questionnaire information were recorded.

### The Edinburgh Postpartum Depression Scale (EPDS)

This questionnaire was developed by Cox et al. to assess postpartum depression status [21]. The Turkish validity and reliability test was performed by Aydin et al. [22]. The scale has 10 self-reported questions, rated on a four-point Likert scale, which are scored from 0 to 3. The scale is scored between 0 and 30 and a cut-off point of 13 or higher is considered as a probable risk for the presence of postpartum depression. Since Cox et al., who developed the EPDS questionnaire, applied the questionnaire to puerperal women at postpartum 6 weeks in their studies, we also applied the EPDS questionnaire at postpartum 6 weeks in this study [21].

### Mother-to-Infant Bonding Scale

The Mother-to-Infant Bonding Scale was described by Taylor et al. [23] and its Turkish translation and validation were performed by Yalcin et al. [24].

The scale consists of eight items, including the emotional states that the mother feels for the baby after the birth. Each item is answered on a four-unit Likert-type rating scale, from “(0) a lot” to “(3) never”. Five of the items indicate negative emotion and are rated in reverse (3–0). A high score indicates a problem in mother-infant attachment. With the “Mother-to-Infant Bonding Scale” developed by Taylor et al., it has been shown that mother-infant bonding develops continuously in the first 12 weeks after birth [23]. Therefore, the scale can be used to assess the immediate postpartum period, as well as to re-evaluate attachment in the near future.

### Statistical analysis

Data analysis was conducted using the IBM Statistical Package for the Social Sciences (SPSS), version 24.0 (IBM Corp., Armonk, NY, USA) and OpenEpi – Open Source Epidemiologic Statistics for Public Health, Version: www.OpenEpi.com [25]. Data are expressed as mean ± standard deviation (SD) or frequency and percentage (%). The suitability of the data for normal distribution was determined by the Shapiro–Wilk test. None of the continuous variables satisfied the assumptions of normal distribution. These variables were compared using Mann–Whitney U test. Categorical variables were compared with the chi-square test or Fisher’s exact test. In the comparison of ordered categorical variables, the Mantel–Haenszel chi-square test for linear trend was used. A two-tailed *p*-value of < 0.05 was considered significant.

For the power calculation, we accepted the post-partum depression prevalence as 14.7% for our population based on the previous literature in the COVID 19 pandemic [14], and the effect size as 0.3. The total sample size of 173 was calculated by G-POWER 3.1.9.7 software with an alpha probability of 0.05 and a power of 0.95.

## Results

The mean age of the subjects included in the study was 27.80 ± 5.24 years. The gestational week was calculated as 39.1 ± 1.21 weeks at the time of delivery. Those with an EPDS questionnaire score of ≥ 13 were considered depressed and those with a score of < 13 were considered as non-depressed, and the cases were divided into two groups. The postpartum depression rate was calculated as 17.4%. Sociodemographic features and the Edinburgh Postpartum Depression Scale (EPDS) and Mother-to-Infant Bonding Scale scores of the groups are given in Table 1. When comparing depressed and non-depressed patients, education level, maternal age, BMI, the Mother-to-Infant Bonding Scale score, history of previous pregnancies, type of delivery (vaginal or cesarean delivery), previous operation history, economic status, employment status and pregnancy follow-up information were found to be similar (*p* > 0.05) (Table 1). It was found that those with postpartum depression were more likely to receive guests at home during the pandemic period compared to those without postpartum depression (29.0% vs 12.2%, *p* = 0.027). The odds of maternal depression for the patients who received guests at home was 3.068 (95%CI [1.149–8.191]) times the odds of patients who did not receive guests at home.

**Table 1:** Sociodemographic features, Edinburgh Postpartum Depression Scale (EPDS) and Mother-to-Infant Bonding Scale scores of the groups

	**Non depressed** (Depression questionnaire score < 13) (n:147)	**Depressed** (Depression questionnaire score ≥ 13) (n:31)	*p*-value
**Educational background**			
Primary School	24 (16.3%)	6 (19.4%)	0.890^a^
Middle School	52 (35.4%)	9 (29%)	
High school	41 (27.9%)	10 (32.3%)	
University	30 (20.4%)	6 (19.4%)	
**Mother's age**	28.1 ± 5.2	26.4 ± 5.4	0.056^b^
	28.0 [24.0; 31.0]	24.0 [22.0; 29.0]	
**Mother's BMI**	26.1 ± 4.2	26.6 ± 4.6	0.663^b^
	25.7 [23.4; 28.3]	25.4 [23.7; 29.6]	
**EDINBURGH POSTPARTUM DEPRESSION SCALE (EPDS) SCORES**	5.1 ± 3.7	16.5 ± 3.5	** < 0.001**^**b**^
	5.0 [2.0; 8.0]	15.0 [14.0; 20.0]	
**MOTHER-TO-INFANT BONDING SCALE SCORES**	0.7 ± 1.1	0.9 ± 1.5	0.287^b^
	0.0 [0.0; 1.0]	1.0 [0.0; 1.0]	
**Pregnancy and birth history**			
How many times has she been pregnant	2.3 ± 1.3	2.3 ± 1.8	0.481^b^
	2.0 [1.0; 3.0]	2.0 [1.0; 2.0]	
How many children did she have	2.0 ± 1.0	1.9 ± 0.9	0.414^b^
	2.0 [1.0; 2.0]	2.0 [1.0; 2.0]	
There is a baby who died after being born	3 (2.0%)	0 (0.0%)	1.000^c^
There is a baby who died in the womb	1 (0.7%)	0 (0.0%)	1.000^c^
**Regarding Current Birth**			
Vaginal birth	64 (43.5%)	13 (41.9%)	0.870^d^
Caesarean birth	83 (56.5%)	18 (58.1%)	
Baby's birth week	39.1 ± 1.2	39.5 ± 1.0	0.061^b^
	39.0 [38.1; 40.0]	40.0 [38.7; 40.1]	
Birth weight of the baby	3275 ± 453	3254 ± 572	0.966^b^
	3306 [2960; 3560]	3200 [2800; 3640]	
**Sex of the infant**			
Female	62 (42.2%)	19 (61.3%)	0.052^d^
Male	85 (57.8%)	12 (38.7%)	
**Operation history**			
Had abdominal surgery	12 (8.2%)	1 (3.2%)	0.084^d^
Had extra-abdominal surgery	8 (5.4%)	5 (16.1%)	
Had no operations	127 (86.4%)	25 (80.6%)	
**Economic status**			
Good	39 (26.5%)	4 (12.9%)	0.077^a^
Middle Class	95 (64.6%)	23 (74.2%)	
Poor	13 (8.8%)	4 (12.9%)	
**Pregnancy follow-ups**			
There is regular follow-up	137 (93.2%)	28 (90.3%)	0.702^c^
**Does she work?**			
Yes	16 (10.9%)	2 (6.5%)	0.743^c^
**Has she received any guests at home?**			
Yes	18 (12.2%)	9 (29.0%)	**0.027**^**c**^

Values are presented as mean ± SD, median [interquartile range] or number (percentage), n; the number of patients in the category

*p*-values were calculated using: ^a^ Extended Mantel–Haenszel chi-square test for linear trend, ^b^ Mann Whitney U test, ^c^ Fisher's Exact Test, ^d^ Pearson Chi-Square test

## Discussion

In this study, the postpartum depression rate was calculated as 17.4%. When depressed (EPDS survey score ≥ 13) and non-depressed (EPDS survey score < 13) patients were compared, education level, maternal age, BMI, Mother-to-Infant Bonding Scale score, history of previous pregnancies, knowledge about the birth, previous operation history, economic status, employment status and pregnancy follow-up information were found to be similar. Mother-to-Infant Bonding Scale scores were found to be similar in depressed and non-depressed patients. It was found that those with postpartum depression were more likely to receive guests at home during the pandemic period than those without postpartum depression. The odds of maternal depression for the patients who received guests at home was 3.068 (95%CI [1.149–8.191]) times the odds of patients who did not receive guests at home.

The rate of postpartum depression varies depending on the pregnant woman's personal traits, the characteristics of the society in which she lives, and extraordinary circumstances such as the pandemic. The prevalence of postpartum depression has been reported as 9% in the prepandemic period [3]. In studies originating from Turkey before the pandemic, the rate of postpartum depression was reported as 13.5% [26], 14% [27], 24% [28], and 28.3% [29] in different studies.

In a meta-analysis evaluating the prevalence and risk factors for postpartum depression in Turkey, the prevalence of postpartum depression was reported as 24%. In the same meta-analysis, mental problems/depression prior to pregnancy, unplanned/unwanted pregnancy, low income/socioeconomic level, bad marital relationship/problems with spouse/dissatisfaction with married life and being a housewife were reported as risk factors for postpartum depression [28]. Among the risk factors in the meta-analysis published in this study, economic status was found to be unrelated to PPD; other risk factors in the meta-analysis were not investigated in our study. For example, Sahin and Seven found a significant relationship between PPD and participants' and husbands’ mean age, duration of the marriage, parity and history of receiving professional psychological support [26]. In this study, similar to the study of Sahin and Seven, EPDS and Mother-to-Infant Bonding Scale were completed in the postpartum 6th week. The relationships between puerperal age, parity variables and PPD, which were found to be statistically significant in the study of Sahin and Seven, were not found to be significant in our study. The prevalence of PPD is different in some studies. This may be due to the fact that the study was conducted in a low-risk group, the difference in inclusion and exclusion criteria and social restrictions during the pandemic period. In addition, the sociodemographic structure of the countries and regions where the studies were carried out may also have an effect on these results.

There are also studies in the literature showing that the prevalence of PPD increased during the COVID-19 pandemic [30]. Zanardo et al. found that postpartum mothers who gave birth during a period of COVID‐19 quarantine presented higher levels of depressive symptoms than mothers in a control group who gave birth during the same period the previous year [31]. Suzuki et al. compared the results of mental screening of postpartum mothers during the COVID‐19 pandemic and mothers during the same period last year and concluded that mother–infant bonding was worse one month after birth among mothers who gave birth during the COVID‐19 pandemic [32]. In a study conducted in China, it was found that the prevalence of perinatal depression increased during the COVID-19 pandemic (26.0% vs 29.6%, *P* = 0.02). They also reported that pregnant women who were underweight, full-time employed, middle income, age < 35 years, primiparous, doing less exercise and not having an appropriate living area appeared to be more susceptible to the outbreak [33]. The current daily number of new COVID-19 cases has been reported as 59,885 in Turkey, 47,136 in Italy, and 65,368 in Japan, and the number of COVID-19 cases in the countries in the studies is similar (https://github.com/CSSEGISandData/COVID-19). Therefore, we think that the increase or decrease in the prevalence of PPD during the pandemic period is not related to the daily number of COVID-19 cases.

In recent studies, the rate of postpartum depression was reported as 34% [33] and 35.4% [34] during the COVID 19 pandemic in the Turkish population. In our study, the rate of postpartum depression during the COVID 19 pandemic was found to be 17.4%. In the study of Arikan et al. in 2015, the rate of postpartum depression was stated as 32.1% in Kutahya province, where the study was done [35]. Compared to the Arikan et al. study conducted in the same region and hospital before the COVID-19 pandemic, the prevalence of PPD appears to have decreased (32.1% vs 17.4%). In our study, similar to the findings of Arikan et al., no difference was found between the mother's age, educational status, employment status and income level, and the frequency of PPD. Unlike previous studies, in our study a significant relationship was found between accepting guests at home and PPD. The odds of maternal depression for patients who received guests at home was 3.068 (95%CI [1.149–8.191]) times the odds of patients who did not receive guests at home. Unlike previous studies, the finding of a significant relationship between accepting guests at home and PPD suggests that not accepting guests may have an effect on the decrease in PPD prevalence during the COVID-19 pandemic. We thought that the lower PPD prevalence than Arikan et al. might be due to the fact that traditional baby celebration ceremonies at home, which can be very crowded and tiring for the new mother, cannot be done because of the pandemic. Accepting guests in the pre-pandemic period, when visitor restriction was not applied, may have added stress and fatigue to the postpartum mother and contributed to depressive symptoms. However, with the current study, it is not possible to conclude that accepting guests is the cause or a risk factor of postpartum depression. Perhaps the postpartum depressed puerperants may have invited people they feel close to because they needed more support.

In other words, accepting guests into the home may not be the cause of postpartum depression, but the result. Unlike other studies in the literature, this study was conducted in a low-risk group. The low prevalence of PPD may also be due to the difference in inclusion and exclusion criteria in the study.

The decrease in the prevalence of PPD during the COVID-19 pandemic period in this study may be related to the characteristics of the society. The society in which the study was conducted is a society that adheres to tiring rituals and traditions such as puerperium celebrations. Relatives and friends of the puerperant woman in rural areas visit the patient from the first hours of the puerperium period in a way that may prevent the patient from resting [36]. During the period of the study, there were legal restrictions on social events (birthday celebrations, etc.) and receiving guests in Turkey and in the province of Kutahya. Possibly, these restrictions may have positively affected the relationship, rest and mental health of postpartum women with their babies.

Because of the restrictions on visits during the pandemic era, postpartum women were able to spend more time with their babies and family, and the rate of postpartum depression may have decreased as a result. Similar to our results, mothers in an Australian study reported spending more time with their babies during the pandemic, resting more, and that the pandemic had positive features like spending more time breastfeeding and connecting with their kids [19, 20]. In a study conducted in a rural area in Turkey during the pre-pandemic period, living in a large family and without the support of the spouse was found to increase maternal postpartum depression 3.53-fold [37]. The study conducted in rural areas in Turkey and the study in Australia showed the effects of the home environment of the puerperant on mental health. These studies support our opinion that the restriction of receiving guests in the rural area during the pandemic period has positive effects on maternal mental health.

In some articles it was claimed that during the pandemic, spouses or relatives of the patient are not escorted to the labor room and that the visitor limitation while in the hospital adds to an increase in postpartum depression rates [38]. In our hospital pregnant women were not allowed to take their spouses to the labor room, but they were allowed to meet with their relatives in the postpartum unit at limited times before the pandemic. One of the reasons why there was no increase in postpartum depression rates according to our results may be that there was no serious change in the physical conditions of the hospital (one-to-one midwifery was not changed) during the pandemic period. Furthermore, our hospital has an adequate number of obstetricians and midwives, and patients in labor can be served with a one-to-one midwife-to-pregnant woman ratio.

The importance of mother-infant attachment was first demonstrated by Klaus and Kennel in 1976 [39]. Mother-infant attachment is important for the physical, cognitive and psychological development of the infant and mother [40]. According to several studies, maternal-infant connections declined during the COVID pandemic period due to heightened levels of worry and sadness [14, 16]. However, there are also studies reporting that maternal-infant attachment was not affected, despite the increase in postpartum depression severity during the COVID 19 pandemic [18–20]. Wilson et al. found that while puerperal women suffered stress as a result of the pandemic, the pandemic period, being alone and constraints had good effects such as mothers spending more time with their newborns, resting more, breastfeeding and connecting with their baby [20]. A study from the United States found that when large groups of visitors stayed in the patient's room for more than 60 min, the mother's communication with the baby was disrupted [41]. Practice of quiet time for postpartum patients (not disturbing the postpartum woman so she can rest for a while) has been shown to increase patient satisfaction, increase breastfeeding and attachment to the baby, and enable healthcare personnel to express their wishes easily [42]. With visitor restrictions during the pandemic era, the mother may have been able to spend more time with the baby, and we believe that the detrimental impact on maternal-infant bonding score can be avoided even in depressed mothers.

Limitations of our study: All factors that could be risk factors for PPD could not be investigated in this study. Therefore, this study may have other unexplored confounders in addition to the factors found to be associated with PPD. Patients were contacted by phone and asked to remember their risk factors, so memory bias may have occurred. Unlike previous studies, the finding of a significant relationship between accepting guests at home and PPD suggests that not accepting guests may have an effect on the decreased prevalence of PPD during the COVID-19 pandemic. However, it is not possible with the current study data to say whether there is a causal relationship between accepting guests in the home and PPD.

## Conclusions

In this study, the postpartum depression rate was calculated as 17.4%. When depressed and non-depressed patients were compared, education level, maternal age, BMI, Mother-to-Infant Bonding Scale score, history of previous pregnancies, information about the birth, operation history, economic status and pregnancy follow-up information were found to be similar. Mother-to-Infant Bonding Scale scores were found to be similar in depressed and non-depressed patients. Although a relationship was found between accepting guests in the postpartum period and postpartum depression, there is a need to investigate with further studies whether there is a causal relationship.

## Data Availability

The datasets generated and/or analysed during the current study are not publicly available [because they contain personal data of the patients and their answers to the applied tests] but are available from the corresponding author on reasonable request.
